# Gray matter contamination in arterial spin labeling white matter perfusion measurements in patients with dementia^☆^

**DOI:** 10.1016/j.nicl.2013.11.003

**Published:** 2013-11-15

**Authors:** Henri J.M.M. Mutsaerts, Edo Richard, Dennis F.R. Heijtel, Matthias J.P. van Osch, Charles B.L.M. Majoie, Aart J. Nederveen

**Affiliations:** a Department of Radiology, Academic Medical Center, Amsterdam, The Netherlands; b Department of Neurology, Academic Medical Center, Amsterdam, The Netherlands; c C.J. Gorter Center for High Field MRI, Department of Radiology, Leiden University Medical Center, Leiden, The Netherlands

**Keywords:** ASL, arterial spin labeling, CBF, cerebral blood flow, CSF, cerebrospinal fluid, GM, gray matter, PSF, point spread function, PV, partial volume, SNR, signal-to-noise ratio, WM, white matter, Arterial spin labeling, Dementia, Gray matter contamination, Partial volume, White matter perfusion

## Abstract

**Introduction:**

White matter (WM) perfusion measurements with arterial spin labeling can be severely contaminated by gray matter (GM) perfusion signal, especially in the elderly. The current study investigates the spatial extent of GM contamination by comparing perfusion signal measured in the WM with signal measured outside the brain.

**Material and methods:**

Four minute 3T pseudo-continuous arterial spin labeling scans were performed in 41 elderly subjects with cognitive impairment. Outward and inward geodesic distance maps were created, based on dilations and erosions of GM and WM masks. For all outward and inward geodesic distances, the mean CBF was calculated and compared.

**Results:**

GM contamination was mainly found in the first 3 subcortical WM voxels and had only minor influence on the deep WM signal (distances 4 to 7 voxels). Perfusion signal in the WM was significantly higher than perfusion signal outside the brain, indicating the presence of WM signal.

**Conclusion:**

These findings indicate that WM perfusion signal can be measured unaffected by GM contamination in elderly patients with cognitive impairment. GM contamination can be avoided by the erosion of WM masks, removing subcortical WM voxels from the analysis. These results should be taken into account when exploring the use of WM perfusion as micro-vascular biomarker.

## Highlights

• A single slice distance analysis was performed.
• Perfusion signal in the white matter was compared with signal outside the brain.
• The application of erosion was compared with removal of partial volume voxels.
• White matter perfusion signal can be distinguished from gray matter contamination.
• The erosion of three voxels is warranted to avoid gray matter contamination.

## 1 Introduction

White matter (WM) perfusion measured with arterial spin labeling (ASL) is a potential in vivo micro-vascular parameter to investigate the interplay between normal aging and degenerative and vascular pathology, such as small vessel disease (Brickman et al., 2009; Zhang et al., 2012). Data on WM perfusion are relatively scarce, because ASL has long been considered unsuitable to measure stable WM cerebral blood flow (CBF) (van Gelderen et al., 2008). Although recent technical advances have enabled these measurements, still a relatively long scan time (10–20 min) is required to capture single voxel WM CBF (van Osch et al., 2009).

Due to the often limited available scan time, clinical investigators either ignore WM perfusion or use it as a reference value (Firbank et al., 2011). Fortunately, voxel-wise comparison of WM perfusion is not always required. It may suffice to average the signal from all WM voxels to provide a single value for the hemodynamic status of the total WM region of interest (ROI). Perfusion signal from such a ROI has recently been shown to be reproducible in elderly patients with dementia (Zhang et al., 2012).

However, contamination of GM signal into WM voxels may seriously affect WM perfusion measurements, because the contrast between GM and WM CBF is large (Pohmann, 2010). Furthermore, changes and correlations are mainly found in GM CBF, while the WM CBF often remains relatively stable (Firbank et al., 2011; Parkes et al., 2004). Therefore, even a fraction of GM contamination may distort WM CBF measurements and its possible clinical correlations.

Main sources of GM contamination are the point spread function (PSF) of the ASL imaging readout module and partial volume (PV) voxels (Petr et al., 2013; van Gelderen et al., 2008). Both have a large effect in ASL due to its low imaging resolution, which is required to compensate for its low signal-to-noise ratio (SNR). Currently, PV voxels are excluded based on the segmentation of a high resolution anatomical scan (Bastos-Leite et al., 2008; Brickman et al., 2009; Zhang et al., 2012). However, simulations indicate that WM voxels without PV may still experience GM contamination due to the PSF (Pohmann, 2010).

Therefore, to correctly interpret perfusion signal averaged from a WM ROI, it is essential to investigate the spatial extent of GM contamination. Can perfusion signal originating from the WM be distinguished from signal blurred from the GM? With this knowledge a WM ROI could be constructed that experiences minimal GM contamination without excluding too many WM voxels. Constructing a WM ROI may be especially challenging in the elderly, because of the decreased T1 and ASL GM-WM contrast and WMH associated with aging (Brickman et al., 2009; Liu et al., 2012; Zhang et al., 2012). The current study investigates the spatial extent of GM contamination in elderly patients with dementia.

## 2 Material and methods

### 2.1 Subject recruitment

41 patients (19 men/22 women, mean age 74.9 ± 9.7 (SD) years) presenting to an outpatient memory clinic were included in this study. Main inclusion criteria were age higher than 18 years and score on the mini-mental state examination equal to or higher than 20. Main exclusion criteria were history of transient ischemic attack or stroke in the last two years or with cognitive decline within three months after the event, major depressive disorder, psychosis or schizophrenia, alcohol abuse, brain tumor, and epilepsy. All patients provided written informed consent and the study was approved by the VU University Medical Center and Academic Medical Center ethical review boards. Of the 41 enrolled participants, 18 fulfilled criteria for mild cognitive impairment and 23 fulfilled criteria for probable Alzheimer's Disease or mixed dementia (Winblad et al., 2004).

### 2.2 MRI protocol

All imaging was performed on a 3.0 T Intera with a SENSE-8-channel head coil and body coil transmission (Philips Healthcare, Best, The Netherlands). To restrict motion the subjects' head was stabilized with foamed material inside the head coils. An isotropic 1 mm 3D T1 weighted scan and 2D FLAIR scan with 3 mm slice thickness were collected using a routine clinical protocol. Added to this protocol was a gradient echo single shot echo-planar imaging pseudo-continuous ASL sequence with the following imaging parameters: resolution, 3 × 3 × 7 mm^3^; FOV, 240 × 240 mm^2^; 17 continuous axial slices; TE/TR, 14/4000 ms; flip angle, 90°; SENSE, 2.5; labeling duration, 1650 ms; post-labeling delay, 1525 ms. Slices were acquired in sequential ascending order. 30 label and control pairs were acquired, resulting in a total scan time of 4 min. Background suppression was implemented with two inversion pulses 1680 and 2830 ms after a pre-labeling saturation pulse. The labeling plane was positioned parallel and 9 cm caudal to the center of the imaging volume (Aslan et al., 2010). For descriptive purposes of the presence of small vessel disease, the Fazekas WM hyperintensity severity scale and four-point global cortical atrophy score were assessed by a trained rater, blinded to the clinical information (Fazekas et al., 1987; Pasquier et al., 1996).

### 2.3 ASL post-processing

Matlab 7.12.0 (The MathWorks, Inc., Natick, MA USA) and the SPM8 toolbox (Statistical Parametric Mapping, Wellcome Trust Centre for Neuroimaging, London, UK) were used for offline data processing with custom-built software. The label and control pairs were pair-wise subtracted after 3D realignment and subsequently averaged to generate perfusion weighted maps. These maps were converted to CBF based on a single compartment model, which assumes that the label remains in the vascular compartment (Wang et al., 2002):

$$\mathrm{CBF}=\frac{6000\mathrm{\lambda \Delta M}e^{\left(\mathrm{TE}/T_{2a}^{*}\right)}}{M_{0a}2\alpha \alpha_{\mathrm{inv}}T_{1a}\left[e^{-w/T_{1a}}-e^{-\left(w+\tau \right)/T_{1a}}\right]}\;\left[\mathrm{mL}/100g/\min\right]$$

where λ is the brain–blood water partition coefficient (0.9 mL/g) (Herscovitch and Raichle, 1985), ΔM is the average difference between control and label for all 30 dynamics, TE is the echo time (14 ms), T_2_*_a_ is the transverse relaxation time of arterial blood (50 ms) (St Lawrence and Wang, 2005), M0_a_ is the equilibrium magnetization of arterial blood, of which an average scanner value was calculated (4.12*10^6^) according to previously described methods (Chalela et al., 2000), α is the assumed pseudo-continuous ASL labeling efficiency (0.85) (Aslan et al., 2010), α_inv_ is the correction for label loss due to background suppression pulses (0.83) (Garcia et al., 2005), T_1a_ is the T_1_ relaxation time of arterial blood (1.650 s) (Zhang et al., 2013), w is the post-labeling delay (1.525 s), τ is the labeling duration (1.650 s). Post-labeling delay differences between slices due to the 2D readout were accounted for. No distinction was made between the quantification of GM and WM voxels. GM and WM probability maps were segmented from the 3D T1 weighted scan and transformed into ASL space by rigid registration of the GM probability map to the perfusion map. Default SPM8 settings were used for segmentation and registration except for the distance between sampling points, which was decreased to 1 mm for increased precision. All CBF maps were scaled such that the mean GM CBF (tissue probabilities > 90%) of each patient matched the population mean (36.8 mL/100 g/min) for the slice used in the distance analysis. Negative values were not excluded. All data analyses were performed in native ASL space to avoid GM contamination due to interpolation.

### 2.4 Distance analysis

**Fig. 1:**
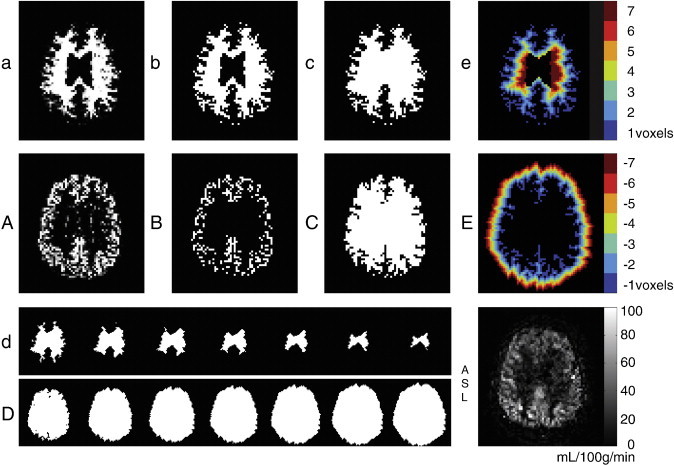
Single slice distance analysis pipeline visualized for a single patient: tissue probability maps (a, A) converted into masks (b, B), gaps filled (c, C), erosion and dilation (d, D) and the resulting city-block geodesic maps (e, E). Lower and upper cases represent WM and GM respectively. In the right lower corner the ASL slice is shown for reference.

Two distance maps were constructed to compare the extent of inward and outward GM contamination. This method enables the comparison between perfusion signal measured in the WM to signal measured outside the brain. Outside the brain, where air or tissue types such as cerebrospinal fluid (CSF), meninges, bone and skin are located, no perfusion signal is expected except from outward GM contamination. This analysis was carried out in 2D and restricted to a single transversal slice (Fig. 1) located 2 slices (14 mm) superior to the basal ganglia. This slice contains a relatively large area of WM, has no central GM and does not experience much distortion or signal dropout as frequently observed anterior in echo-planar imaging. The procedures of the distance analysis are stepwise listed here, and visualized in Fig. 1.

1) The WM probability map (a) was converted into a WM mask (b), including tissue probabilities > 10%. This low probability threshold avoids the exclusion of WM hyperintensity voxels, which are frequently misclassified as GM voxels. Subsequently, the GM probability map (A) was converted into a GM mask (B), including tissue probabilities > 90%, which is complementary to the WM mask at the GM/WM boundary.
2) Any remaining regions inside the WM or GM masks (such as CSF) were masked as well (c and C), such that erosions or dilations affected the outer borders of the masks only.
3) Erosions were applied to the WM mask (d) and dilations to the GM mask (D), using a cross structural element with radius 1.
4) Inward (e) and outward (E) city-block geodesic distance maps were created by labeling each voxel for number of erosions required to remove this voxel from the WM mask (e) or for the number of dilations required to add this voxel to the GM mask (E).

Consequently, the resulting distance maps show for each WM voxel its shortest distance (in voxels) to the outer border of the WM mask (Fig. 1e) and for each voxel outside the brain its shortest distance to the outer border of the GM mask (Fig. 1E). Since the in-plane voxel size is 3 × 3 mm, a distance of 1 voxel presents a distance of 3 mm. All voxels with the same distance were projected on the CBF maps, to compute the mean CBF and voxel count for each distance.

### 2.5 Partial volume analysis

To investigate the influence of PV voxels, the same WM tissue probability map as used for the distance analysis was converted to multiple binary masks with WM tissue probabilities ranging from 80% to 100% with a bin size of 1% (e.g. 80–81%, 81–82%, etc.). This range was selected, as it encloses probability thresholds that have been previously selected in WM research (Brickman et al., 2009; van Osch et al., 2009; Zhang et al., 2012). These WM masks were projected on the ASL data and their mean WM CBF, GM-WM CBF ratio and voxel count were calculated. For both the distance and PV analysis, the individual mean GM CBF (tissue probabilities > 90%) was defined as GM CBF. This GM CBF was also used to calculate the GM-WM ratio for the inward distances 1 to 7 voxels.

## 3 Results

**Fig. 2:**
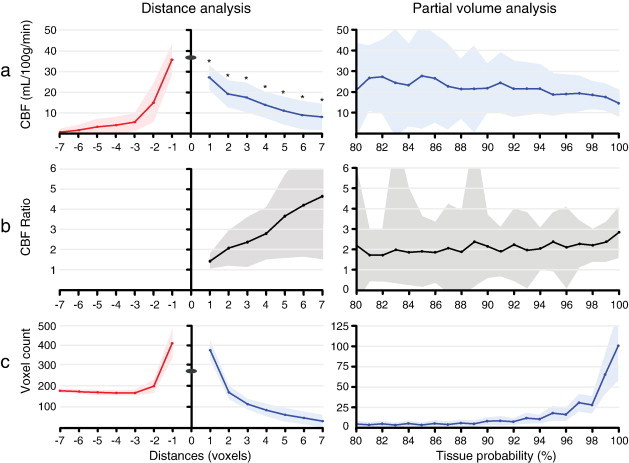
a–c show single slice distance analysis (left column) and partial volume analysis (right column). a) mean CBF; b) mean GM-WM CBF ratio; c) mean number of voxels. The distance numbers on the left x-axis correspond with distances in Fig. 1. Distances − 1 to − 7 represent GM mask dilation steps, distances 1 to 7 represent WM mask erosion steps. Distance 0 represents the mean GM CBF (tissue probabilities > 90%). The numbers on the right x-axis represent bins of the WM tissue probabilities (bin size 1%). Lines and planes represent mean values and ± 95% CI respectively. Significant differences (*p* < 0.001) between negative and positive distances are indicated by an asterisk (*).

Patient characteristics are summarized in Table 1. The mean GM CBF was 36.8 ± 8.5 mL/100 g/min. Outward GM contamination was mainly observed in the first three voxels (distances − 1 to − 3 voxels), whereas distances − 4 to − 7 voxels showed very low signal (Fig. 2a). The inward decrease of WM signal was smaller than the outward signal decrease (*p* < 0.001 with paired sample Student's *t*-test, indicated with asterisks in Table 2 and Fig. 2a). In the PV analysis, the WM CBF and GM-WM ratio seemed to show decreasing GM contamination with increasing tissue probabilities (Fig. 2a).

**Fig. 3:**
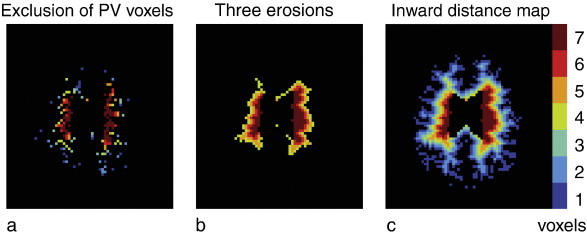
a–c visualize the difference between two masks obtained by either a) the exclusion of partial volume (PV) voxels by thresholding the WM mask at a tissue probability of 100% or b) the application of three erosions on a large WM mask (tissue probabilities > 10%). The same WM distance map color scale (Fig. 1c, reprinted here for reference (c)) is applied here to visualize the position of the voxels included in both masks.

A comparison of the left and right graphs of Fig. 2a–b shows the relation of GM contamination with the inclusion of voxels containing 80 – 100% WM PV. Mean CBF and GM-WM CBF ratio of tissue probabilities 80 to 99% can be compared with distances 1 to 3 voxels (*p* = 0.728 independent sample *t*-test). The WM CBF and GM-WM CBF ratio at 100% WM tissue probability (i.e. WM voxels without PV) can be compared with distance 4 voxels (*p* = 0.810). At higher inward distances (5–7) the mean CBF decreased further and reached lower values than with the exclusion of all PV voxels (tissue probability 100%) (*p* < 0.001). Similarly, at these higher distances the GM-WM CBF ratio reached higher values than with the exclusion of all voxels containing < 100% WM PV. Fig. 3 shows the difference between a WM mask without these voxels (tissue probability = 100% without erosions) and a WM mask with these voxels (tissue probabilities > 10%) but with three erosions applied. It illustrates that the exclusion of voxels containing < 100% WM PV did not remove all subcortical WM voxels whereas it did remove voxels within the deep WM.

## 4 Discussion

The findings of this study are threefold. Firstly, the outward GM contamination suggests that GM contamination mainly affects the first three subcortical WM voxels and has only minor influence on deep WM signal, beyond three voxels distance from the GM. Secondly, the significant asymmetry between the inward and outward signal indicates that the detected signal within the WM voxels reflects WM perfusion signal. Finally, Fig. 3 indicates that GM contamination is not restricted to voxels that contain more than 0% GM PV. These results provide insight in the distinction of PSF from the effect of PV voxels, and show that, within a WM ROI, WM signal can be separated from the contamination of GM signal.

Using probabilistic tissue segmentation, generally two different methods can be applied to avoid GM contamination (van Osch et al., 2009; Zhang et al., 2012). The tissue probability threshold can be set high to exclude all voxels containing less than 100% WM PV. Alternatively, it can be set relatively low (e.g. only excluding < 10% WM PV) in combination with a number of erosions applied on the outside of the mask. Here, we have compared the two methods. With an increase in excluded voxels that contain GM or CSF PV, we observed decreasing GM contamination, a trend that is in agreement with previous findings (van Osch et al., 2009). As the WM CBF and GM-WM CBF ratio at 100% tissue probability (i.e. only voxels containing 100% WM PV) were comparable to CBF and GM-WM CBF ratio at a distance of 4 voxels, it appears that it would suffice to exclude all voxels containing less than 100% WM PV. However, Fig. 3a shows that 100% WM voxels also resided within the subcortical WM, where GM contamination was observed. In addition, the segmentation algorithm removed voxels within the deep WM, where no GM contamination was observed. The removal of deep WM voxels is probably the result of segmentation errors due to WM hyperintensities or CSF PV voxels (Chen et al., 2011). Although CSF contamination decreases the measured WM CBF, this effect includes only noise and does not bias clinical correlations — as is the case for GM contamination. Therefore, we conclude that the application of erosion on the outer boundary of a WM mask is a more effective way to avoid GM contamination compared to the exclusion of voxels containing less than 100% WM PV.

The GM-WM ratio has been frequently used to compare perfusion results independent from global quantification differences. Nevertheless, discrepancies exist between literature values of this ratio, even within modalities. Where some authors have reported ratios between 2 and 3, others reported ratios between 4 and 6 (Pohmann, 2010; Rempp et al., 1994; Xu et al., 2010). Whereas studies with the highest values were focused on deep WM or used methods that were less sensitive to GM contamination, studies with lower values seem to have employed a larger ROI or lower imaging resolution (Pohmann, 2010; van Gelderen et al., 2008). Our ratios in the deep WM (distances 4–7 voxels) are within the range of the first whereas our ratios in subcortical WM (distances 1–3 voxels) are more comparable to the latter. In addition, the ratios in subcortical WM are comparable to those obtained in the PV analysis (80–99%). This adds to the point that the exclusion of voxels containing less than 100% WM PV may not suffice to avoid GM contamination.

Our ratios in deep WM, on the other hand, are still slightly lower than previously reported values. This may be attributable to aging or WMH (Brickman et al., 2009; Liu et al., 2012). Alternatively, these ratios may depend on quantification differences between GM and WM CBF, such as the T_1_ relaxation time of tissue, blood–brain partition coefficient or tissue arrival times. In the current study, we aimed to visualize the distance analysis in CBF units without influencing our results by differences in CBF quantification. Therefore, an identical model was applied for the quantification of GM and WM CBF and the label was assumed to remain in the vascular compartment (Parkes, 2005). This assumption may especially be valid in the elderly, because of their prolonged transit times (Liu et al., 2012). Moreover, such a simple model eliminates PV effects introduced by quantification based on T1 segmentation, due to the possibility of registration mismatches. Alternatively, tissue probability maps can be acquired using the same ASL readout module, which enables separate GM- and WM-quantification that is not affected by registration mismatches (Petr et al., 2013). In the current study, these mismatches may be increased by echo-planar imaging distortions in regions that are close to air-tissue transitions, which are predominantly GM areas (Deichmann et al., 2002). This highlights the importance of proper registration between the T1 and the ASL scan.

It should be acknowledged that the design of the current analysis is based on segmentations of an anatomical 3D T1 scan, and assumes homogeneous perfusion values across all voxels with the same distance from the GM-WM boundary. This assumption is required to average multiple voxels for sufficient SNR. Whether or not perfusion is homogeneous across WM is currently unknown. On the other hand, it is well known that transit times differ within the WM (Pohmann, 2010). This heterogeneity has probably contributed to the continuing CBF decrease from distances 4 to 7 voxels, where no GM contamination is expected (as shown in Table 2 and Fig. 2a). Alternatively, this may be caused by CBF decreasing lesions, such as WM hyperintensities, or CSF contamination (Brickman et al., 2009). Outside the brain, the measured signal may not entirely be dependent upon the PSF. Factors that may have contributed to the signal found outside the GM include extra-cranial vessels, perfusion of the skin and motion artifacts.

The heterogeneity of acquisition details that determine the PSF, such as the ASL readout resolution, readout time or T2* blurring, may limit the extrapolation of the present results to other studies. One previous study simulated the effect of PSF in a single large central WM voxel on multiple spatial resolutions, assuming a GM and WM CBF of 80 and 0 mL/100 g/min, respectively. Whereas on a low isotropic resolution such as 12.5 mm a contamination of 10 mL/100 g/min could be measured in the central WM, on an isotropic resolution of 3.1 mm (which is in-plane comparable to our acquisition) only 0.08 mL/100 g/min GM contamination was left (Pohmann, 2010). This simulation is in line with the present results, which demonstrate that perfusion measured in deep WM contains only minor GM contamination. Furthermore, the PSF differs between 2D and 3D readouts.

The current distance analysis was restricted to a single slice to compare the 2D in-plane PSF versus the effect of PV voxels. This is a valid comparison for 2D readout modules, since they have no PSF in the through-plane direction — except for crosstalk from slice profile, which is negligible in slices as thick as 7 mm. Although 3D readouts exhibit increased SNR and improved background suppression allowing for higher spatial resolution, they experience increased GM contamination due to their wider 3D PSF — especially in the through-plane direction (Vidorreta et al., 2012). Even though methods exist that numerically correct this GM-WM contamination, a 2D readout module can be preferred when uncontaminated WM CBF measurements are more important than spatial or temporal SNR (Asllani et al., 2008; Vidorreta et al., 2012).

To summarize, these data illustrate that, using pseudo-continuous ASL, WM perfusion signal can be distinguished from GM contamination within clinically feasible scan time in patients with cognitive impairment. Because of the PSF, GM contamination is not restricted to PV voxels and it seems necessary to apply erosion to remove subcortical WM voxels. It is expected that this method would only work in some slices, as for the majority of slices too few or no WM voxels will be left after 3 erosions. Whether this is sufficient for clinical studies should be clarified in further research. These results should be taken into account when exploring the use of WM perfusion as micro-vascular biomarker.

## Figures and Tables

**Table 1:** Clinical and radiological characteristics (*n* = 41).

Age (years)	74.9 (9.7)
Gender (male/female)	19/22
Mini-mental state examination	24.9(2.9)
Geriatric depression scale	2.6(2.1)

*Fazekas*	
0	4%
1	44%
2	15%
3	37%

*Global cortical atrophy*	
1	19%
2	59%
3	22%

Of continuous variables the mean is shown. Standard deviations are shown in parentheses. Findings of categorical variables are presented in percentages. Mini-mental state examination ranges from 0 to 30 (higher score equates with better cognitive function) and the geriatric depression scale ranges from 0 to 15 (higher score equates with more symptoms of depression).

**Table 2:** Outward and inward GM contamination (*n* = 41).

Distance	CBF	Distance	CBF	GM-WM ratio
(voxels)	(mL/100 g/min)	(voxels)	(mL/100 g/min)	
− 1	35.7 ± 7.4	+ 1	27.3 ± 5.8 *	1.4 ± 0.3
− 2	15.2 ± 9.3	+ 2	19.4 ± 6.4 *	2.0 ± 0.7
− 3	6.0 ± 4.3	+ 3	17.7 ± 7.0 *	2.3 ± 1.0
− 4	4.5 ± 4.1	+ 4	14.2 ± 6.2 *	4.1 ± 2.8
− 5	3.7 ± 3.9	+ 5	11.5 ± 6.7 *	4.1 ± 2.2
− 6	2.1 ± 2.6	+ 6	9.3 ± 6.8 *	5.0 ± 3.4
− 7	1.2 ± 1.7	+ 7	8.4 ± 6.3 *	4.9 ± 3.0

Mean ± standard deviation of CBF are shown for distances − 1 to − 7, representing outward GM contamination (left columns) and for distances 1 to 7, representing inward GM contamination (right columns). The GM-WM ratio for the inward distances is shown as well. Significant differences (*p* < 0.001) between negative and positive distances are indicated by an asterisk (*).
